# Work for the College of Nursing?

**Published:** 1916-05-27

**Authors:** 


### THE EDITOR'S LETTER-BOX.

### Work for the College of Nursing?

*To the Editor of* The Hospital.

Sir,—In response to the invitation from the Editor on page 152 of The Hospital for the 13th inst., I beg to offer the following points for the consideration of the Council of the College of Nursing, and of matrons, sisters, and nurses generally.

1. At the outset, discussion will necessarily arise as to what modifications in and new methods of training, if any, would tend to improve the present system of training schools in Great Britain.

2. Another important matter is the extent to which it is desirable to encourage the establishment of preliminary training schools where accepted candidates for training from groups of hospitals might receive, say, thirteen weeks' preliminary instruction before beginning work in the wards of a great hospital. If each University town had a preliminary training school of this type, it should not be difficult for each such school to obtain lecturers of competence and repute. Each such school might successfully handle 200 pupils a year, in four batches of fifty pupils each. It is held that most probationers could provide a fee of £12 for the course. It is possible that a little organisation would readily produce offers of scholarships, not only for nurse-pupils, but for the more accomplished nurse graduates from each school.

(*a*) One ground for securing the establishment of an adequate number of preliminary training centres is that they would tend to foster a spirit of comradeship between the nurses of the different hospitals, and so might lead to lifelong friendships of real importance.

(*b*) There is another point. In the thirteen weeks entirely given up to the Theory of Nursing, each pupil should receive instruction in elementary anatomy, physiology, and hygiène, bed-making, invalid cookery, preparation of dressings, poultice-making, and temperature-taking.

3. A further important matter is the provision of graduate training schools where certificated nurses, willing to specially train in the duties of a matron, of a ward or other sister, and any special field of nursing, could spend six months in equipping themselves practically for the discharge of the duties of the selected office. Some, at any rate, of these graduate training schools should provide courses similar to those given at a teachers' training college in practical dispensing, dietetics, domestic economy, and other fields allied to nursing, accompanied by facilities for visiting tuberculosis dispensaries, school clinics, and any special centre of nursing activities in the immediate neighbourhood of each graduate school. In this way, in a reasonably short time, these specially trained graduate nurses would bring with them to the hospitals where they may be promoted to responsible posts system and knowledge, which could not fail to have substantial influence for good in their new field of work. In this connection the Council of the College will no doubt make itself thoroughly familiar with the excellent system which has been found to work so well in the building up and perfecting of the Queen's Jubilee Institute for Nurses.

I have merely set forth the above as suggestions, in order, as I hope, to initiate a discussion in The Hospital on the present needs of the nursing profession in the directions indicated, and to elucidate points including the suggestion of plans by which these needs could be most efficiently and promptly supplied. I am, yours faithfully, A Hospital Matron.

### Withholding the Annual Report: An Explanation Asked For.

*To the Editor of* The Hospital.

